# High-Flow Nasal Cannula vs. Continuous Positive Airway Pressure Therapy for the Treatment of Children <2 Years With Mild to Moderate Respiratory Failure Due to Pneumonia

**DOI:** 10.3389/fped.2020.590906

**Published:** 2020-11-13

**Authors:** Cong Liu, Wei Yu Cheng, Jun Shao Li, Tian Tang, Ping Li Tan, Lin Yang

**Affiliations:** ^1^Department of Infectious Diseases, Children's Hospital of Chongqing Medical University, Chongqing, China; ^2^National Clinical Research Center for Child Health and Disorders, Chongqing Medical University, Chongqing, China; ^3^Ministry of Education Key Laboratory of Child Development and Disorders, Chongqing Medical University, Chongqing, China; ^4^Chongqing Key Laboratory of Pediatrics, Chongqing Medical University, Chongqing, China; ^5^Department of Emergency, Children's Hospital of Chongqing Medical University, Chongqing, China

**Keywords:** CPAP, HFNC, pneumonia, mild to moderate respiratory failure, randomized controlled study

## Abstract

**Background:** The aim of this prospective randomized controlled study was to further compare the clinical benefits and adverse reactions of HFNC with CPAP in the treatment of mild to moderate respiratory failure due to pneumonia in children below 2 years old.

**Methods:** Using a prospective randomized controlled study method, 84 patients with pneumonia and mild to moderate respiratory failure admitted to the Children's Hospital Affiliated to Chongqing Medical University from January 2018 to December 2019 were randomly divided into the HFNC group and the CPAP group. It was registered as a clinical trial at clinical trials.gov, registration number: ChiCTR2000030463.

**Results:** The analyses included 84 patients. No differences were observed between the two groups in baseline demographic or physiological characteristics. Treatment failure necessitating intubation and transfer to the PICU was noted in six of 43 infants (14%) in the HFNC group, as compared with four of 41 infants (10%) in the CPAP group (*P* > 0.05). There were no significant differences between the two groups in the duration of hospital stay, the duration of non-invasive respiratory support, and mortality. The 10 infants who experienced treatment failure had more severe hypoxemia with lower PaO2/FiO2 (HFNC 182 ± 11.5 and CPAP 172 ± 8.6). We found that both the HFNC group and the CPAP group showed significantly improved oxygenation and relief of respiratory distress after treatment. No differences were observed between the two groups in the development improvement of RR, PaO2, PaCO2, SpO2, and PH. Assessment of the occurrence of adverse events showed that the HFNC group had a lower level of nasal injury, a lower risk of abdominal distension, a lower intensity and frequency of sedation, and better tolerance.

**Conclusion:** HFNC is an effective and safe initial respiratory support treatment in children <2 years with mild to moderate respiratory failure due to pneumonia, and the incidence of intubation and death is very low; concurrently, the comfort and tolerance of HFNC are better. To some extent, HFNC is a well-tolerated alternative to CPAP.

## Introduction

Pneumonia is a major cause of respiratory failure in pediatric patients (1), It's the world's leading infectious cause of death in children younger than five, causing 808,694 deaths, or 15% of all deaths in this age group, in 2017 (2, 3).

Hypoxemia is the main risk factor leading to pneumonia-related death in children (4); conventional oxygen therapy is limited to delivering high concentration oxygen through standard nasal intubation to treat hypoxemia. The hallmark of severe pneumonia is respiratory failure resulting in hypoxemia, increased work of breathing, and/or hypercarbia, and all of these conditions respond to the provision of positive pressure. Although continuous positive airway pressure (CPAP) is currently an effective and safe non-invasive respiratory support model in bronchiolitis (5), the availability of CPAP is limited because of the requirement of technical skills, clinical, equipment, and maintenance follow-up (6).

High-flow nasal cannula (HFNC) oxygen therapy has been increasingly used in children of respiratory support, and it is easy to use and is well-tolerated by patients. It can regulate the oxygen flow and concentration, and it provides excellent humidification (7–9) and has some CPAP effect. In recent years, a few large sample-sized prospective clinical studies and meta-analysis have suggested that HFNC has a positive effect on oxygenation improvement, decreased work of breathing, intubation rate reduction, and need for respiratory support after extubation (10). HFNC has been found to be better than standard oxygen therapy, oxygen delivered through a standard nasal cannula, at a rate of up to 2 l of 100% oxygen per minute, to treat hypoxemia (11, 12); furthermore, it has been shown to have better tolerance and less adverse reactions than CPAP (13). HFNC is associated with a lower 90-days mortality rate, and intubation rate in severe hypoxemic patients treatment with high-flow oxygen, standard oxygen, or non-invasive ventilation(NIV) did not result in significantly different (14). HFNC is a new method of providing respiratory support in newborns, infants, children, and adults (9, 15–17), and it may be considered as a potential first-line strategy for the management of acute respiratory failure.

However, there are few randomized controlled trials (RCTs) comparing HFNC with CPAP in children with pneumonia and respiratory failure. A recent meta-analysis of HFNC vs. nasal continuous positive airway pressure (nCPAP) in children with respiratory distress revealed that nCPAP is associated with a lower risk of treatment failure than HFNC in infants aged 1–6 months with acute lower respiratory infection (ALRI), moderateto-severe respiratory distress, and severe hypoxemia, but there was inadequate conclusive evidence in infants aged 6–12 months (18). However, only 2 trials in this article compared HFNC with nCPAP in patients with severe pneumonia, and there is a lack of trials in patients with mild to moderate respiratory failure. Therefore, it is still unclear whether HFNC can be an effective and pleasant alternative to CPAP in children with mild to moderate respiratory failure.

Based on this uncertainty, we focused on children <2 years with mild to moderate respiratory failure due to pneumonia, and we performed this prospective RCT to further evaluate the clinical benefits and adverse reactions of HFNC in comparison with CPAP for the treatment of children <2 years with mild to moderate respiratory failure due to pneumonia(Chinese Clinical Trial Registry registration number: ChiCTR2000030463).

## Materials and Methods

### Patients

We performed a prospective RCT of two respiratory support models for children with mild to moderate respiratory failure due to pneumonia in Chongqing, China.

The clinical diagnostic criteria of pneumonia are acute infection of lung parenchyma and / or pulmonary interstitium, causing varying degrees of hypoxia and infection symptoms, usually accompanied by fever, cough, shortness of breath, lung moist rales, abnormal chest X changes (19).The imaging evidence of pneumonia is consolidation (with or without dense or fluffy opacity of bronchography), other infiltration (linear and patchy alveolar or interstitial density), or pleural effusion. Severe pneumonia is characterized by cyanosis, shortness of breath, RR ≥ 70 per min (infant) or RR ≥ 50 per min (over 1 year old), assisted breathing (groan, nasal fan, trigeminal sign), intermittent apnea or oxygen saturation <92%. Patients were treated with CPAP or HFNC therapy in addition to standard management of severe pneumonia.

Inclusion criteria were as follows: (1) children <2 years with severe pneumonia, who have no indication of emergency tracheal intubation and have relatively stable vital signs under traditional oxygen inhalation.

Mild to moderate respiratory failure defined by hypoxemia level of 150 < oxygenation index (PaO2/FiO2 ratio) < 300 and PaCO2 <70 mm Hg with spontaneous breathing under standard oxygen (14); (2) family members have signed the informed consent form and approval of the ethics committee of the hospital has been obtained.

Exclusion criteria were as follows: (1) patients with complicated congenital heart disease, severe malnutrition, neuromuscular disease, metabolic disease, and other serious basic diseases; patients with chronic lung disease, secondary respiratory failure, including bronchopulmonary dysplasia, congenital airway dysplasia, and other chronic lung diseases; (2) patients who stopped treatment in the middle as the withdrawal standard, and cure or death as the termination standard.

### Procedures

The study was conducted between January 2018 and December 2019 in the emergency ward of Children's Hospital Affiliated to Chongqing Medical University.

Patients were randomized as soon as study eligibility was confirmed.

Eligible patients were randomized to either CPAP or HFNC (1:1) using sequentially numbered envelopes. The study was not blinded, since HFNC and CPAP are already used in practice and are recognizable by clinicians. The HFNC group: patients received Airvo2 type warm humidification high flow double chamber nasal oxygen therapy ventilator (Fisher Parker company of New Zealand) for ventilation within 3 h. The initial parameter was set at 50-60% oxygen concentration, and the inhaled oxygen flow was set at 2 L/kg/min to a limit of 20 L/min to maintain the transcutaneous oxygen saturation ≥92–94%.

The CPAP group: the initial parameter was set at 50–60% oxygen concentration, the pressure was set at 4–6 cm H_2_O, and the flow rate of oxygen supply was set at 5–10 L/min to maintain the transcutaneous oxygen saturation ≥92–94%.

The vital signs were monitored closely, and the improvement in respiration, heart rate, and SpO2 was evaluated 1 h later. If improvement was found, the treatment was continued. If the SpO2 level was lower than 92% during the treatment, and there was persistent tachycardia and tachypnea, tracheal intubation and invasive ventilator- assisted ventilation were performed. The FiO2 level was adjusted according to the PaO2 and SpO2 levels to provide an oxygen level as low as possible to maintain SpO2 of at least 92% (or≥94%). When the FiO2 level was ≤0.3 and the SpO2 level was ≥94%, tachycardia and tachypnea were obviously relieved; HFNC and CPAP were discontinued during general low-flow oxygen therapy at a flow rate of 1–2 L/min.

### Observations and Paraclinical Examination

All patients were monitored for arterial blood gas analysis at the time of admission, and then at least once a day for 2 days. Chest x-ray was performed prior to treatment, and the diagnosis of pneumonia was confirmed.

All patients were intensively observed by a trained nurse and doctor. The RR, HR, PaCO2, PaO2, SpO2, and PaO2/FiO2 ratio were observed before treatment and 1, 24, and 48 h after treatment.

Interruption mainly occurred when the child's condition improved. Treatment failure was comprehensively evaluated by a responsible physician to determine whether the patient should be transferred to PICU for mechanical ventilation treatment.

### Outcomes

Primary outcomes, including the incidence of treatment failure and intubation, the duration of hospital stay and PICU stay, the duration of non-invasive respiratory support, and mortality, were compared between the two groups.

Secondary effectiveness outcomes were adverse reactions and change in HR, RR, SpO2, PaO2, and PaCO2 at 1, 24, and 48 h after the initiation of treatment.

Adverse reactions mainly included children's comfort and tolerance to ventilation measures, the need for sedatives, observation of abdominal distension, pneumothorax, facial compression, cardiac arrest, and other adverse reactions.

### Statistical Analysis

Previous studies have found that the failure rate of nCPAP and HFNC in infants with moderate to severe bronchiolitis is 31.0 and 50.7%, respectively (6). We hypothesized that the failure rate would be similar for pneumonia with respiratory failure (30% and 50%) so as to meet the criterion for non-inferiority; considering an α error of 0.05, a β error of 0.20, and a sampling rate of 0.9, a sample size of 68 patients was calculated.

For group comparisons, according to the normal distribution, the measurement data were represented by median (interquartile range) or mean ± standard deviation, and Wilcoxon rank-sum test or two-sided Student's *t*-test was used for non-normally distributed data and normally distributed data, respectively. Count data were represented by rate, and Chi2 test was used for binary outcomes.

For all analyses, the significance level was defined as *p* < 0.05. All statistical analyses were performed using SPSS 23.0 statistical software.

### Ethics

The study was prospectively approved by the Ethics Regional Committee of Children's Hospital Affiliated to Chongqing Medical University, and it was registered as a clinical trial at clinical trials.gov, registration number:

ChiCTR2000030463. Written informed consent was obtained from all legal guardians prior to performing any study-related procedures.

## Results

**Figure 1:**
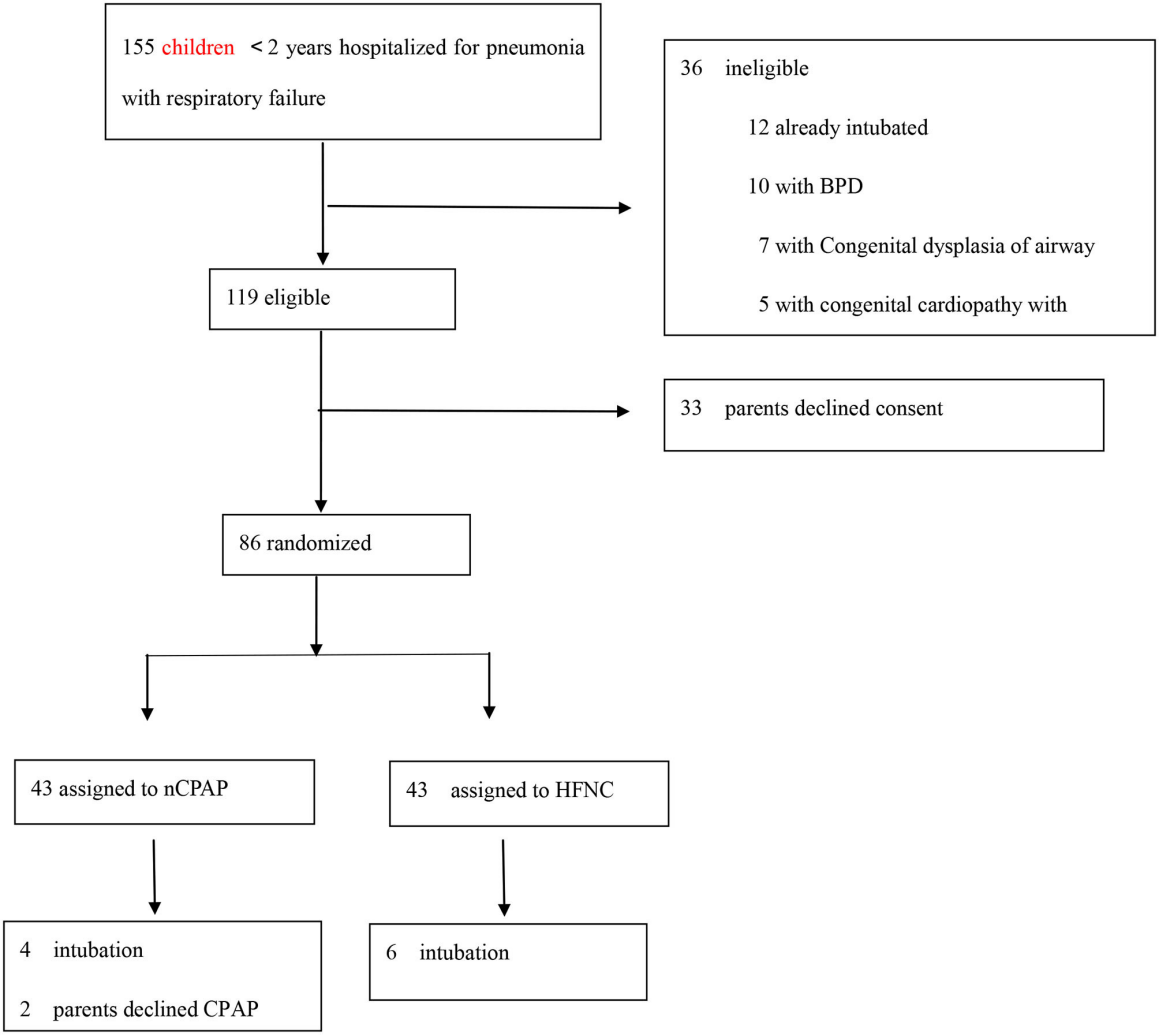
Number of children screened, assigned to a trial group, and preliminarily analyzed.

During the study period, a total of 155 infants aged <2 years were hospitalized for pneumonia with respiratory failure and were assessed for eligibility. Figure 1 describes the process of screening, assigned to the trial group, and the number of children. A total of 71 children were excluded. Thus, 84 children were included in the analysis (Figure 1).

Table 1 describes the 84 children included in the study. No differences in baseline demographic or physiological characteristics were observed between the two groups (Table 1).

**Table 1:** Baseline characteristics of children with pneumonia and mild to moderate respiratory failure.

	**HFNC (43)**	**CPAP (41)**	***P*-value**
**CHARACTERISTIC**			
Age, months, median (range)	3 (2–11)	4 (1–11)	*p* = 0.993
**Distribution—no. (%)** ≤12 mo	36 (84)	32 (78)	
>12 mo	7 (16)	9 (22)	
Weight, kg, median (range)	6 (5–9)	6.5 (4.5–9.25)	*p* = 0.911
Female sex *n* (%)	19 (44)	18 (43)	*p* = 0.979
Symptom duration, days, median (range)	7 (4–8)	5 (4–8.5)	*p* = 0.414
HR, min, mean (sd)	153.6 ± 17.1	147 ± 15.5	*p* = 0.111
RR, min, mean (sd)	59.8 ± 7.4	57.2 ± 9.2	*p* = 0.151
PaO2, mmHg, mean (sd)	54.3 ± 7.8	53.3 ± 9.1	*p* = 0.584
PaCO2, mmHg, median (range)	42 (37–49)	42 (38–48)	*p* = 0.661
SPO2, % median (range)	90 (88–91)	90 (87–91)	*p* = 0.856
PaO2 / FiO2, mean (sd)	217.1 ± 31.1	213.1 ± 36.2	*p* = 0.584
PH, mean (sd)	7.38 ± 0.06	7.38 ± 0.07	*p* = 0.895
Imaging diagnosis, *n* (%)	43 (100)	41 (100)	—
Congenital heart disease, *n* (%)	5 (12)	7 (17)	*p* = 0.476
Viral cause, *n* (%)	28 (65)	20 (49)	*p* = 0.130

*Comparison of general data on baseline characteristics between the two groups[cases (%). Values are expressed as mean (SD) ±SD or numbers (%),No differences were found between the two groups*.

### Primary Outcome

Treatment failure necessitating intubation and transfer to the PICU was noted in 6 of 43 infants (14%) in the HFNC group, as compared with 4 of 41 infants (10%) in the CPAP group (*P* > 0.05) (Table 2). There were no significant differences between the two groups in the duration of hospital stay, the duration of non-invasive respiratory support, or treatment failure with escalation of oxygen therapy (*P* > 0.05) (Table 2).

**Table 2:** Primary outcomes in the study groups.

**Variable**	**HFNC group (43)**	**CPAP group (41)**	***P*-value**
**PRIMARY OUTCOMES**			
Duration of stay in the hospital, days	8 (7–9)	8(7–9)	*p* = 0.461
Duration of non-invasive respiratory support, days	2 (2–3)	3 (2–3)	*p* = 0.090
Transfer to the ICU, no. (%)	6 (14)	4 (10)	*p* = 0.553
Intubation, no. (%)	6 (14)	4 (10)	*p* = 0.553
Mortality, no. (%)	0	0	—
Use of sedatives, no. (%)	17 (40)	34 (83)	*p* = 0.000
Adverse events, no. (%)	2 (5)	11 (27)	*p* = 0.005
Serious adverse event	0	0	—
Abdominal distension, no. (%)	2 (5)	7 (17)	*p* = 0.066
Pneumothorax	0	0	—
Cardiac arrest	0	0	—
Respiratory arrest	0	0	—
Apnea episodes	0	0	—
Skin lesions, no. (%)	0	4 (10)	*p* = 0.036

*Comparison of primary outcomes between the two groups [cases (%)]. Values are expressed as mean (SD) ±SD or numbers (%)*.

The mortality of the two groups was the same. CPAP was not superior to HFNC in the treatment of pneumonia with respiratory failure with respect to mortality, escalation of oxygen therapy, and Length of stay (LOS) or PICU admission rate. The children in the CPAP group who experienced treatment failure were older [8 months (range, 2.25–12.25 months)] than those in the HFNC group [2 months (range, 1–5.25 months)], and the absolute heart rate and respiratory rate were lower in the CPAP group. The severity of disease as measured on admission was similar in the two trial groups with respect to the PaO2 (HFNC 45.5 ± 2.9 mmHg vs. CPAP 43.0 ± 2.1 mmHg), SPO2 [HFNC 88% (range, 85.75–90.25) vs. CPAP 87% (range, 85.5–88.5)] and a lower PaO2/FiO2 level (HFNC 182 ± 11.5 vs. CPAP 172 ± 8.6); and this suggested that the degree of respiratory failure and hypoxemia of who experienced treatment failure was more serious than that at baseline of this group. There were no significant between-group differences in the duration of hospital stay and the duration of non-invasive respiratory support, but the duration of stay in the ICU was significantly lower in the HFNC group than in the CPAP group (Table 3).

**Table 3:** Characteristics of the 10 children who experienced treatment failure necessitating intubation.

**Characteristic**	**HFNC group (6)**	**CPAP group (4)**	***P*-value**
Age, months, median (range)	2 (1–5.25)	8 (2.25–12.25)	0.279
Weight, kg, median (range)	5.3 (4.47–6.98)	8.5 (4.38–9.25)	0.52
Symptom duration, days, median (range)	7.0 (6.25–7.25)	5 (5–11.75)	0.504
HR, min, mean (sd)	164 ± 9.4	142 ± 7.4	0.005
RR, min, mean (sd)	57.5 ± 7.3	48.2 ± 6.8	0.078
PaO2, mmHg, mean (sd)	45.5 ± 2.9	43.0 ± 2.1	0.180
PaCO2, mmHg, median (range)	42.5 (37.5–45.75)	44 (41.25–56.5)	0.52
SPO2, % median (range)	88 (85.75–90.25)	87 (85.5–88.5)	0.589
PaO2/FiO2, mean (sd)	182 ± 11.5	172 ± 8.6	0.180
Duration of stay in the hospital–days	15 (11.5–18.25)	19 (15.5–21.75)	0.165
Duration of stay in the ICU–days	9 (6.75–10.75)	15 (10.5–19.5)	0.042
Duration of non-invasive respiratory support–days	2 (1.75–2)	2 (1.25–2)	0.759

*Comparison of general data on treatment failure between the two groups [cases (%)]. Values are expressed as mean (SD) ±SD or numbers (%)*.

### Secondary Outcomes

The rate of adverse events in the HFNC group (5%) was significantly lower than that in the CPAP group (27%) (*P* < 0.05).Two of 43 infants (5%) in the HFNC group experienced abdominal distension compared with 7 of 41 (17%) in the CPAP group (*P* > 0.05). In addition, 4 of 41 infants (10%) in the CPAP group developed nasal mucosal injury and none of the infants in the HFNC group developed this type of injury. No serious life-threatening adverse events were observed in both groups, including pneumothorax, cardiac and respiratory arrest, and asphyxia (Table 2). In this study, we found that the use of sedatives was less with HFNC than CPAP. The proportion of usage of sedatives, including chloral hydrate, midazolam, and phenobarbital sodium, was 39.5% in the HFNC group and 82.9% in the CPAP group. Further, 45% of children in the CPAP group needed more than one sedative compared with 21% of children in the HFNC group (*P* = 0.00). The HFNC group was better in terms of lactation, and there was no significant change in the coordination of sucking, swallowing, and breathing. HFNC was well-tolerated without any obvious adverse effects (Table 2).

After 24 h of oxygen therapy PaO2 and SpO2 levels in both the HFNC and CPAP groups were significantly improved (*P* < 0.05), but there was no significant difference between the two groups. After oxygen therapy, the heart rate and respiratory rate in the two groups were slightly lower than those before oxygen therapy, and respiratory distress was relieved (Table 4). At 24 and 48 h after admission, reduction in respiratory distress and improvement in blood gas parameters were noted in the two groups.

**Table 4:** Secondary outcomes in the study groups.

**Variable**	**HFNC group (43)**	**CPAP group (41)**	***P*-value**
**RR, min, mean (sd)**			
T0	59.8 ± 7.4	57.2 ± 9.2	*p* = 0.151
T1	54.2 ± 7.7^*^	54.02 ± 6.7	*p* = 0.896
T24	51.9 ± 8.0^*^	52.2 ± 5.7^*^	*p* = 0.899
T48	51.5 ± 8.2^*^	51.5 ± 5.7^*^	*p* = 0.987
**HR, min, mean (sd)**			
T0	153.6 ± 17.1	147 ± 15.5	*p* = 0.111
T1	148.7 ± 13.4	143.2 ± 10.0	*p* = 0.037
T24	144.4 ± 13.2 ^&^	139.1 ± 11.2 ^&^	*p* = 0.049
T48	138.5 ± 14.4 ^&^	137.1 ± 10.3 ^&^	*p* = 0.537
**SpO2, % median (range)**			
T0	90 (88–91)	90 (87–91)	*p* = 0.856
T1	95 (94–96) ^#^	95 (94–96) ^#^	*p* = 0.462
T24	95 (94–96) ^#^	96 (95–97) ^#^	*p* = 0.144
T48	95 (94–96) ^#^	96 (95–97) ^#^	*p* = 0.105
**PaO2, mmHg, mean (sd)**			
T0	54.3 ± 7.8	53.2 ± 9.1	*p* = 0.420
T24	89 (75–107) ^@^	87 (76–112) ^@^	*p* = 0.907
T48	91.5 (84.7–97.5) ^@^	96 (81–122.25) ^@^	*p* = 0.217
**PaCO2, mmHg, mean (sd)**			
T0	42 (37–49)	42 (38–48)	*p* = 0.661
T24	41 (35–46)	42 (37.5–45)	*p* = 0.707
T48	40.5 (35–46)	41 (38–46)	*p* = 0.615
**PH, mean (sd)**			
T0	7.38 ± 0.06	7.38 ± 0.07	*p* = 0.895
T24	7.40 (7.35–7.43)	7.37 (7.34–7.42)	*p* = 0.233
T48	7.38 (7.34–7.45)	7.38 (7.35–7.42)	*p* = 0.612

*Improvement in the mean respiratory rate (RR), heart rate (HR), SpO2, PaO2, PaCO2, and PH during 48h of respiratory support. Values are expressed as mean (SD) ±SD or numbers (%)*.

* *RR*,

& *HR*,

# *SpO2*,

@ *PaO2, compare with T0 p < 0.05*.

At the same time, the oxygen concentration decreased gradually and the SpO2 level tended to be stable, but there was no significant difference between the two groups. Blood gas analysis and monitoring showed that PaO2, PaCO2, pH, and oxygen saturation were significantly improved after oxygen therapy, but there was no difference between the two groups (Table 4).

## Discussion

HFNC is a relatively effective and safe non-invasive ventilation model in the pediatric ward, Emergency Department, and PICU (13, 19). It was recently accepted as a treatment option for noninvasive ventilation or before endotracheal intubation (14, 20–22). A recent systematic review and meta-analysis that included 9 RCTs also stated that HFNC may decrease the need for tracheal intubation without causing any impact on the mortality in patients with acute hypoxemic respiratory failure (23).

In this RCT involving children with pneumonia and mild to moderate respiratory failure, in terms of the primary outcomes, there was no significant difference in the intubation rate, admission rate of the PICU, the duration of hospital stay, and need for invasive respiratory support in the HFNC group compared to the CPAP group. Vahlkvist et al. also reported that no differences were observed in improvement of respiratory rate or PaCO2 between the HFNC group and the CPAP group in infants with bronchiolitis. Treatment failure was rare in both groups, and there was no significant difference in the length of hospitalization or treatment duration (10).

To our knowledge, only few studies have compared HFNC and CPAP in the management of pneumonia in children. In a multicentre pilot RCT, Padmanabhan et al. found that intubation required within 72 h in HFNC patients was slightly higher than that in the CPAP group, but the difference was not statistically significant. Length of hospital stay and PICU stay and duration of invasive ventilation showed no significant difference. The HFNC group had fewer ventilator-free days at day 28, and most of the adverse events reported were mild or moderate in the CPAP group (11). An open RCT of bubble CPAP for children with severe pneumonia and hypoxemia in Bangladesh also showed that there was no difference in treatment failure between patients in the bubble CPAP group and the HFNC group (24). A review of initial non-invasive oxygenation strategies in patients with *de novo* acute hypoxemic respiratory failure included 16 studies, and it found that non-invasive ventilation (NIV) was associated with a significant reduction in intubation rates compared with conventional oxygen therapy, but there was no significant difference in the efficacy with HFNC (25). These findings are consistent with our research results, but the results are different from those of the meta-analysis of HFNC vs. CPAP in children with respiratory distress (18). It was found that CPAP may be superior to HFNC in the treatment moderate to severe respiratory distress and severe hypoxemia in infants aged 1–6 months, but no significant difference was observed between the two groups in infants aged 6–12 months.

The differences between our study and that study can be explained by the difference in the patient population. Our study specifically aimed at children <2 years with mild to moderate respiratory failure due to pneumonia. We included pneumonia with an oxygenation index (PaO2/FiO2)>150, which excludes moderate to severe respiratory failure, infants with severe accompanying symptoms, basic diseases, and infants with immediate respiratory support and intubation. This may explain some of the differences in results, and it may indicate that HFNC is mainly an alternative tool for the treatment of pneumonia with mild to moderate respiratory failure.

At the same time, we also observed that 10 children who experienced treatment failure and were treated with intubation had severe hypoxemia, lower PaO2/FiO2 (HFNC 182 ± 11.5 and CPAP 172 ± 8.6) and severe respiratory distress. Although the symptoms were relieved after HFNC treatment, the outcome did not change to a large extent, and this therapy could not reduce the risk of tracheal intubation and transfer to the PICU. Consistent with other studies, HFNC has a role in the treatment of moderate or moderate to severe respiratory failure, but the risk of treatment failure is also increased. A PaO2/FiO2 < 200 was a strong predictor of intubation under NIV (26). Therefore, a systematic review suggested that HFNC may be superior to conventional oxygen therapy in acute hypoxemic respiratory failure patients, and it may be reasonable to consider HFNC as an intermediate level of oxygen therapy between conventional oxygen therapy and NIV (16).

Our study showed that HFNC is a relatively safe, well-tolerated, and effective non-invasive respiratory support model in children, and its action mechanism may be related to flushing of the dead cavity of the nasopharynx, increasing the lung compliance, and providing low-level of positive airway pressure (17, 27). Milesi confirmed that in infants with bronchiolitis, HFNC flow ≥2 L/kg/min was associated with the production of a mean pharyngeal pressure ≥4 cmH2O (28, 29), and they also stated that HFNC flow 3 L/kg/min did not reduce the risk of failure compared with HFNC flow 2 L/kg/min (29).

Therefore, in our study, the HFNC flow rate was set at 2 ml/Kg/min, the average pharyngeal pressure was set at ≥4 cm H_2_O, and the CPAP pressure was set at 4–6 cm H_2_O, which was equivalent to the positive end expiratory pressure induced by HFNC. Thus, it could be explained that the efficiency of respiratory support for mild to moderate respiratory failure was equivalent, there was improvement in oxygenation and relief of respiratory distress, and the intubation rate and duration of respiratory support were similar.

Our study has certain limitations. It was a single center study and the number of patients was limited. Therefore, we need to perform large multicenter studies with adequate statistical power to explore the findings of this study.

In conclusion, this RCT involving infantile pneumonia with mild to moderate respiratory failure demonstrated that HFNC is an effective initial respiratory support treatment in the early stage of hospitalization, the use of preemptive respiratory support is safe, and the incidence of intubation and death is very low; concurrently, the comfort and tolerance of HFNC are better. To some extent, it is a well-tolerated alternative to CPAP.

## Data Availability Statement

The raw data supporting the conclusions of this article will be made available by the authors, without undue reservation.

## Ethics Statement

The studies involving human participants were reviewed and approved by Ethics Regional Committee of Children's Hospital Affiliated to Chongqing Medical University. Written informed consent to participate in this study was provided by the participants' legal guardian/next of kin. Written informed consent was obtained from the minor(s)' legal guardian/next of kin for the publication of any potentially identifiable images or data included in this article.

## Author Contributions

LY and PT contributed to the conception and design of this study. WC and CL organized the data and performed the statistical analysis. TT and JL drafted the manuscript. LY and CL wrote sections of the manuscript. All authors contributed to manuscript revision, read, and approved the submitted version.

## Conflict of Interest

The authors declare that the research was conducted in the absence of any commercial or financial relationships that could be construed as a potential conflict of interest.
